# Clinicopathological significance and prognostic implications of Ube2v1 expression in colorectal cancer

**DOI:** 10.3892/mi.2023.119

**Published:** 2023-10-31

**Authors:** Qiang Ma, Jin Bao, Naiying Sun, Xingjie Yang, Li Liu, Ying Chen, Wenjun Guo, Lixiang Gao

**Affiliations:** 1 Department of Pathology, Sunshine Union Hospital, Weifang, Shandong 261000, P.R. China; 2 Department of Pathology, Fangzi District People's Hospital, Weifang, Shandong 261200, P.R. China

**Keywords:** ubiquitin-conjugating enzyme E2 variant 1, colorectal cancer, immunohistochemistry, clinicopathology

## Abstract

The present study aimed to investigate the expression of ubiquitin-conjugating enzyme E2 variant 1 (Ube2v1) in colorectal cancer (CRC) and its clinical significance. The differential expression of Ube2v1 in CRC tissues and normal intestinal tissues, as well as the association between Ube2v1 expression and the prognosis of patients with CRC were analyzed using bioinformatics analyses. TIMER database analysis revealed higher Ube2v1 expression in CRC tissues than in normal intestinal tissues. Cancerous and normal tissues collected retrospectively from 37 cases of CRC between July, 2022 and June, 2023 were analyzed for Ube2v1 expression using immunohistochemistry, and the associations between Ube2v1 expression and the clinical pathological features of patients with CRC were analyzed. Ube2v1 expression was associated with lymph node metastasis in patients with CRC (P<0.05). However, bioinformatics analysis using the GEPIA2 and HPA database revealed that Ube2v1 was not associated with the overall survival of patients with CRC. On the whole, the present study demonstrates that due to its high expression and association with lymph node metastasis, Ube2v1 may serve as a potential target for the treatment of CRC.

## Introduction

Colorectal cancer (CRC) is one of the most common malignant tumors and is the second leading cause of cancer-associated mortality worldwide (1); it is becoming increasingly prevalent in younger individuals (2). Screening and early detection can significantly reduce the mortality rate associated with CRC. However, even following tumor resection and systemic treatment, the 5-year survival rate is only 40% for patients without tumor metastasis and ~20% for those with metastatic CRC (3), and the quality of life of these patients is markedly reduced. Currently, the etiology of CRC development is not yet fully understood. Therefore, it is critical to identify molecular targets that can lead to the development of novel therapeutic approaches for patients with CRC.

Ubiquitination is a post-translational modification that involves the covalent attachment of ubiquitin to target proteins. Ubiquitination relies on ubiquitin-activating enzymes (E1s), ubiquitin-conjugating enzymes (E2s), ubiquitin-ligase enzymes (E3s), the 26S proteasome, and de-ubiquitinating enzymes (4,5). Ubiquitination mediated by the ubiquitin-proteasome system is essential for maintaining cellular protein homeostasis. The binding of ubiquitin to substrates typically involves three key steps: The initiation step catalyzed by E1, the intermediate step of covalently attaching ubiquitin to E2, and the final step of transferring ubiquitin from E2 to the protein substrate, usually facilitated by E3(6). Protein degradation controlled by the ubiquitin system removes misfolded proteins and plays a critical role in regulating cellular signal transduction (7,8). Ubiquitin-dependent protein degradation mediated by the ubiquitin-proteasome system regulates the cell cycle, DNA repair, immune function and other cellular processes (7).

Members of the E2 family are key components of the ubiquitin-proteasome system and play roles in the development of malignant tumors, including CRC. Takahashi *et al* (9) found that the ubiquitin-conjugating enzyme E2 C (Ube2C) gene was highly expressed in 50% of patients with CRC and that Ube2C played a significant role in the liver metastasis of advanced-stage CRC. Ubiquitin-conjugating enzyme E2 variant 1 (Ube2v1), also known as Uev1A, is a mammalian homolog of yeast MMS2 and an auxiliary factor of the ubiquitin-conjugating enzyme, Ube2n (10). Ube2v1 is a unique sub-member of the E2 family as it lacks the conserved catalytic cysteine in E2(11). Ube2v1 complexes with Ube2n and activates the nuclear factor κB (NF-κB) signaling pathway, which is involved in the regulation of cancer (12). Previous studies have demonstrated that Ube2v1 activates the NF-κB signaling pathway via the Ube2v1-Ubc13 complex and promotes CRC metastasis by epigenetically suppressing autophagy (6,13). However, the clinical and pathological relevance of Ube2v1 in CRC has not yet been elucidated, at least to the best of our knowledge. The present study thus examined Ube2v1 expression in CRC tissues, and identified associations between Ube2v1 expression and the clinical and pathological characteristics of patients with CRC in order to determine the clinical significance of Ube2v1 expression in this type of cancer.

## Patients and methods

### Study population

Patients with CRC who underwent surgical treatment at the Sunshine Union Hospital (Weifang, China) from July, 2022 to June, 2023 were selected retrospectively and their cancer tissues were collected. The inclusion criteria were as follows: i) Patients who were confirmed to have CRC by a colonoscopy biopsy and post-operative pathological analysis; ii) patients who did had not received any pre-operative radiotherapy, chemotherapy or other related treatments; iii) all specimens were fixed promptly, processed correctly, and met the standards for slide preparation in which the cancer tissues contained all the layers of the tumor; and iv) the patient medical records were complete. The exclusion criterion was a history of malignant tumor treatment in other parts of the body. The inclusion of clinical data was based on written informed consent provided by the patient and approval from the Ethics Committee of Sunshine Union Hospital (Approval no. 2022-04-0043). A total of 37 cases were included, with 19 males and 18 females. The ages of the patients ranged from 43-80 years, with a median age of 63 years. There were 12 patients <60 years of age and 25 patients ≥60 years of age. There were 20 cases of CRC in the colon and 17 cases of CRC in the rectum. There were 20 cases with tumor diameters <5 cm and 17 cases with tumor diameters ≥5 cm. There were 20 cases with moderate to high pathological differentiation and 17 cases with poor differentiation. There were 9 cases with invasion depths of T1-T2 and 28 cases with invasion depths of T3-T4. There were 6 cases with vascular invasion and 31 cases with no vascular invasion. There were 7 cases with perineural invasion and 30 cases with no perineural invasion. There were 16 cases with lymph node metastasis and 21 cases with no lymph node metastasis. The clinicopathological characteristics of the patients are presented in Table I.

### Bioinformatics analysis

The ‘Diff Exp’ module in the TIMER database (https://cistrome.shinyapps.io/timer/) (14) shows differentially expressed genes in various cancers and normal tissues. TIMER was utilized to predict differences in Ube2v1 expression between cancer and normal tissues from multiple cancer patients. The association between Ube2v1 expression and the prognosis of patients with CRC was also investigated using the Gene Expression Profiling Interactive Analysis (GEPIA)2 database (http://gepia2.cancer-pku.cn) and the Human Protein Atlas database (HPA, https://www.proteinatlas.org/).

### Immunohistochemical staining and evaluation

CRC specimens were fixed in 10% neutral formalin, embedded in paraffin, and cut into 3-µm-thick sections for immunohistochemical staining. The sections were incubated with the primary antibody Ube2v1 (cat. no. E-AB-18501, Elabscience Biotechnology Co., Ltd.; diluted 1:100) overnight at 4˚C. Subsequent procedures were performed according to the enhanced polymer method (Data S1), and finally, the slides were sealed with neutral balsam. The results of immunohistochemistry were evaluated by two independent pathologists. The staining intensity was scored on a scale of 0-3 with 0 indicating negative staining, 1 indicating weak staining, 2 indicating medium staining, and 3 indicating strong staining. The extent of staining, which was defined as the percent of the tumor that stained positive relative to the whole tumor, was scored on a scale of 0-4 with 0 indicating 0%, 1 indicating 1-25%, 2 indicating 26-50%, 3 indicating 51-75%, and 4 indicating 76-100%. An overall protein expression score ranging from 0-12 was calculated by multiplying the staining intensity and staining extent scores (6). Overall scores <6 indicated a low expression, and scores ≥6 indicated a high expression.

### Statistical analysis

Statistical analyses were performed using SPSS version 26.0 software (IBM Corp.). The two-sided Chi-squared (χ^2^) test was used to evaluate significance of the associations between Ube2v1 expression and the clinicopathological characteristics of the patients with CRC. Fisher's exact test was used when >20% of the cells in the contingency table had an expected count of ≤5 individuals. P<0.05 was considered to indicate a statistically significant difference.

## Results

### Ube2v1 expression is higher in CRC tissues

**Figure 1:**
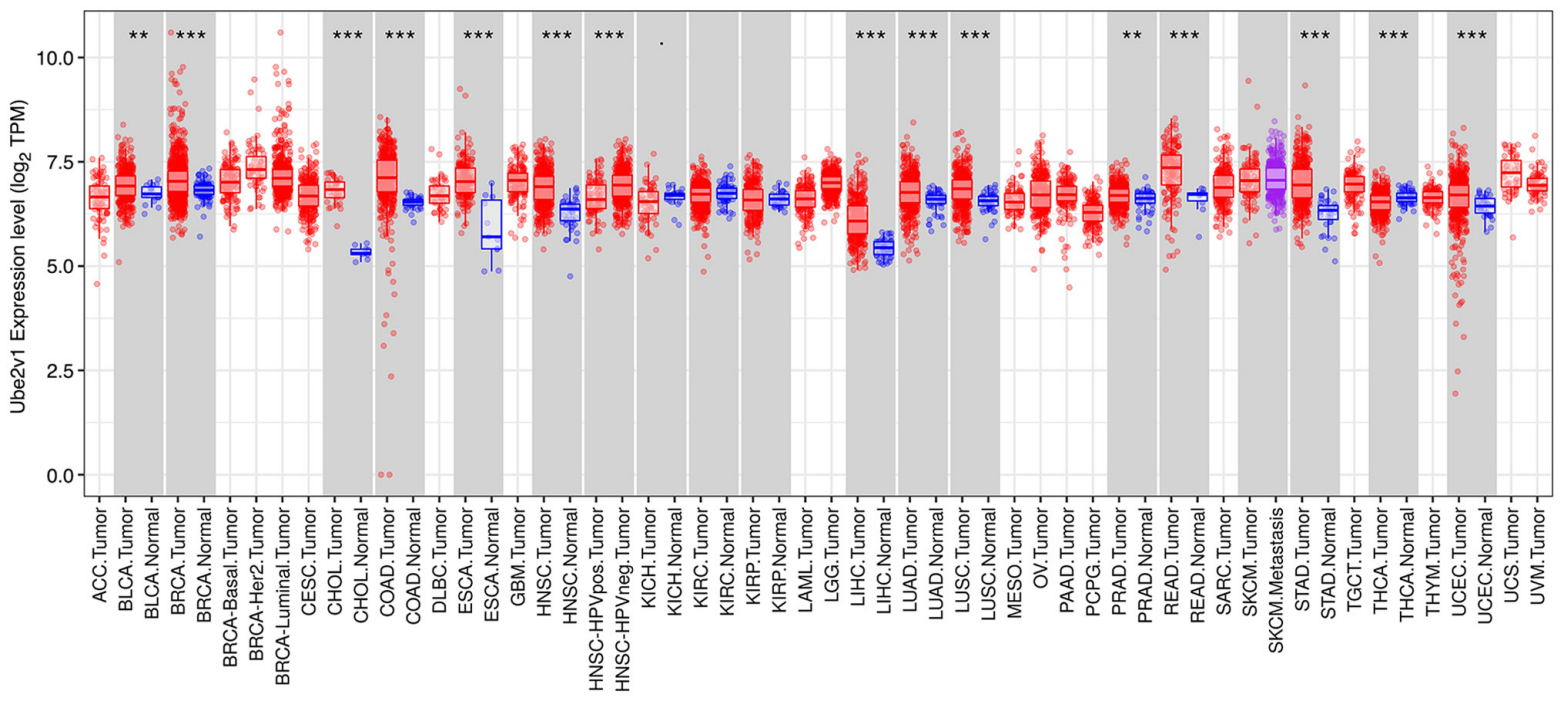
Expression of Ube2v1 in various types of tumors. The analysis using the TIMER database revealed the differential expression of Ube2v1 between various types of tumors, including colorectal cancer and normal tissues. ^**^P<0.01 and ^***^P<0.001. Ube2v1, ubiquitin-conjugating enzyme E2 variant 1; ACC, adrenocortical carcinoma; BLCA, bladder Urothelial Carcinoma; BRCA, breast invasive carcinoma; CESC, cervical squamous cell carcinoma and endocervical adenocarcinoma; CHOL, cholangiocarcinoma; COAD, colon adenocarcinoma; DLBC, lymphoid neoplasm diffuse large B-cell lymphoma; ESCA, esophageal carcinoma; GBM, glioblastoma; HNSC, head and neck squamous cell carcinoma; HPV, human papillomavirus; KICH, kidney chromophobe; KIRC, kidney renal clear cell carcinoma; KIRP, kidney renal papillary cell carcinoma; LAML, acute myeloid leukemia; LGG, brain lower grade glioma; LIHC, liver hepatocellular carcinoma; LUAD, lung adenocarcinoma; LUSC, lung squamous cell carcinoma; MESO, mesothelioma; OV, ovarian serous cystadenocarcinoma; PAAD, pancreatic adenocarcinoma; PCPG, pheochromocytoma and paraganglioma; PRAD, prostate adenocarcinoma; READ, rectal adenocarcinoma; SARC, sarcoma; SKCM, skin cutaneous melanoma; STAD, stomach adenocarcinoma; TGCT, testicular germ cell tumors; THCA, thyroid carcinoma; THYM, thymoma; UCEC, uterine corpus endometrial carcinoma; UCS, uterine carcinosarcoma; UVM, uveal melanoma.

The distributions of gene expression levels are displayed using box plots in the TIMER database results, with the statistical significance of differential expression evaluated using the Wilcoxon test. The results showed that Ube2v1 expression was significantly elevated in colon adenocarcinoma and rectum adenocarcinoma tissues compared to normal tissues (Fig. 1).

### Associations between Ube2v1 expression and clinical pathological features of patients with CRC

**Figure 2:**
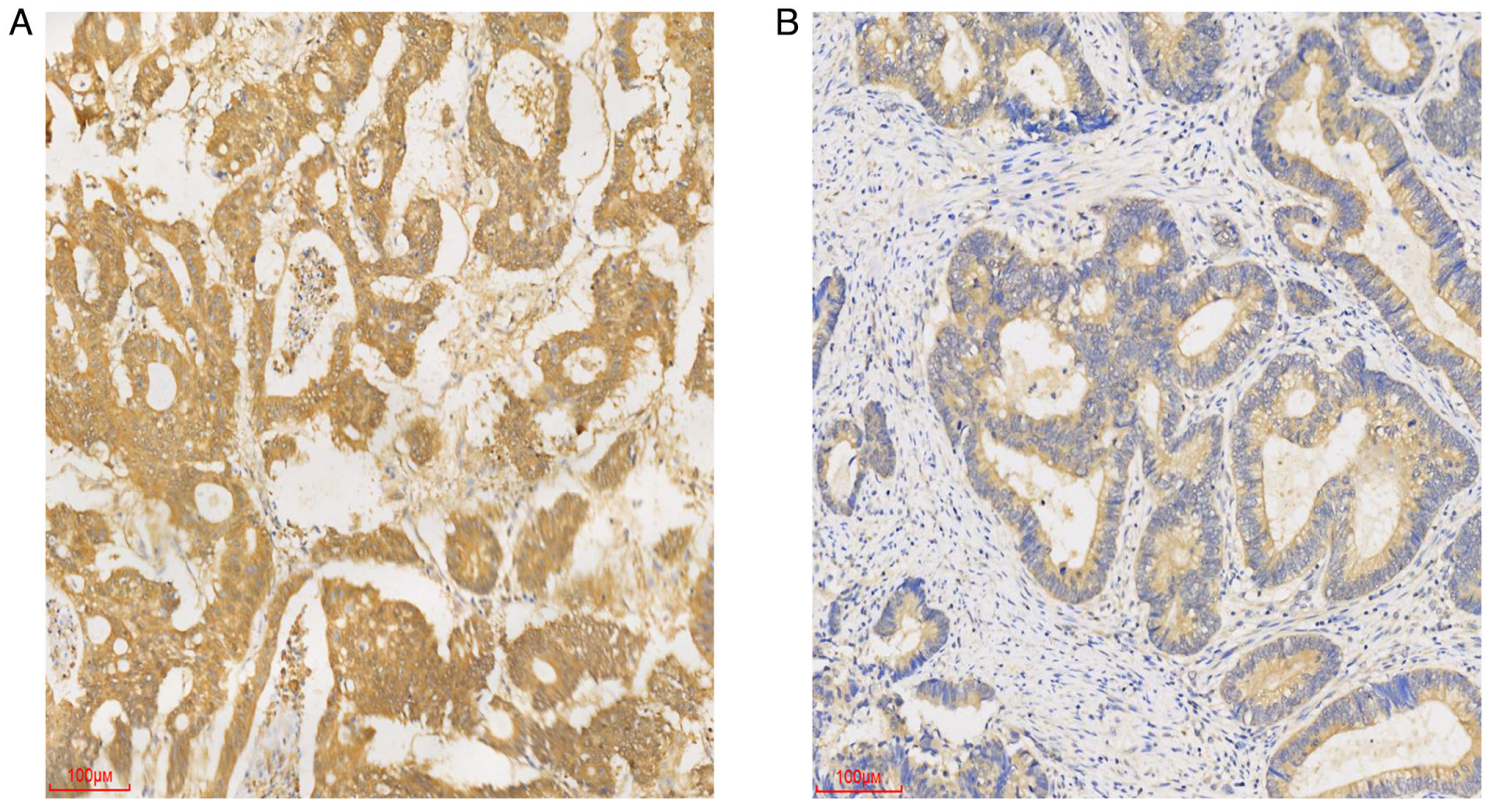
Immunohistochemical staining illustrating (A) strong positive staining in cancer cells, and (B) weak positive staining in cancer cells (magnification, x100; scale bar, 100 µm).

Ube2v1 expression in CRC tissues was evaluated using immunohistochemistry, and the associations between Ube2v1 expression and the clinical features of patients with CRC, including sex, age, tumor location, tumor size, degree of differentiation, depth of tumor, vascular invasion, perineural invasion and lymph node metastasis were analyzed. Immunohistochemical staining revealed that Ube2v1 expression in CRC tissues localized to the cytoplasm (Fig. 2). In addition, the Ube2v1 expression level was closely associated with the presence of lymph node metastasis and exhibited a trend towards stage/invasion association (pT stage) (Table I).

### Ube2v1 expression is not associated with the prognosis of patients with CRC

**Figure 3:**
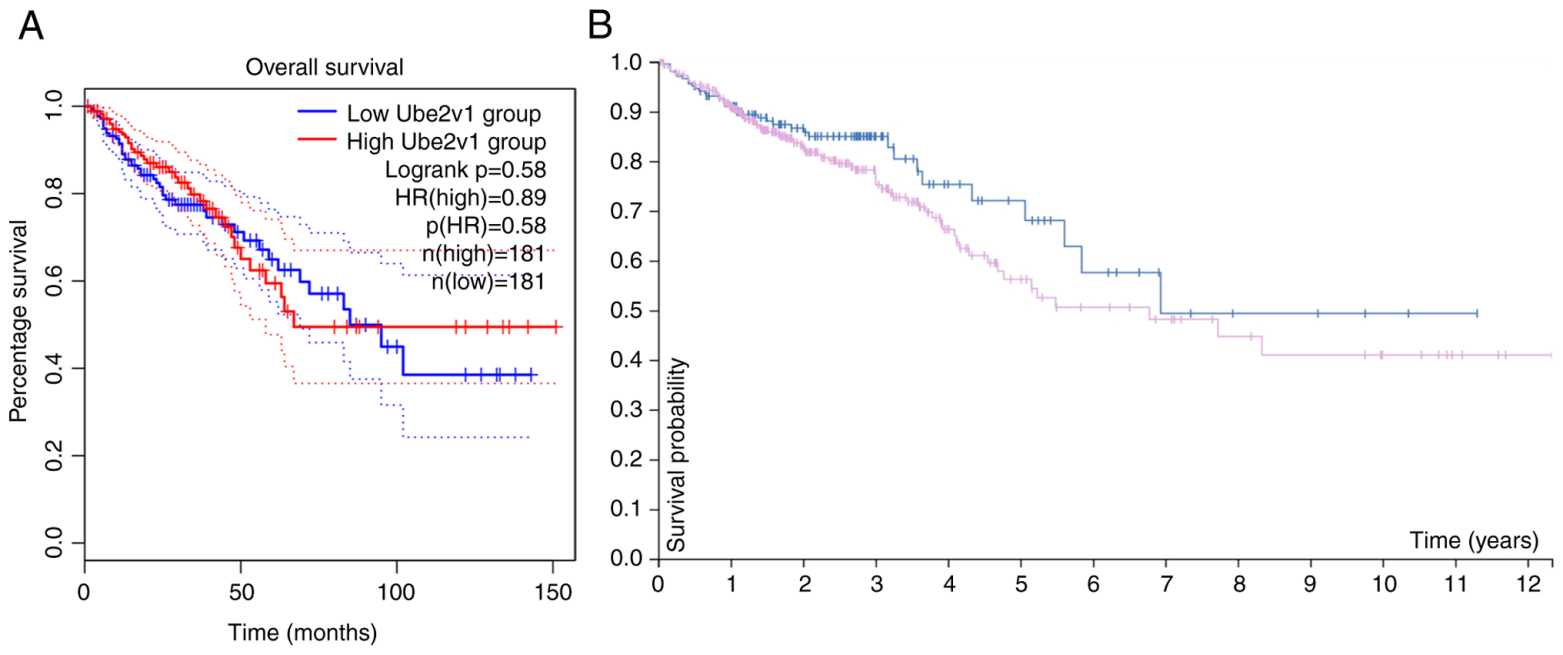
Survival analysis using the (A) GEPIA2 and (B) HPA database. The results indicated that the expression of Ube2v1 was not associated with the prognosis of patients with colorectal cancer. Ube2v1, ubiquitin-conjugating enzyme E2 variant 1.

The association between Ube2v1 expression level and patient prognosis was assessed using log-rank tests in both the GEPIA2 and HPA databases. The analysis of the GEPIA2 database (P=0.58, Fig. 3A) and the HPA database (P=0.15, Fig. 3B) did not reveal any significant association between the expression level of Ube2v1 and the prognosis/survival of patients with CRC.

## Discussion

Ube2v1 is an E2 variant and the corresponding gene is located on chromosome 20q13.2(15). Ube2v1 was initially identified as an activator of the trans-activating factor c-fos, and it is the mammalian homolog of yeast MMS2(16). However, Ube2v1 and MMS2 have different functions. For example, MMS2 forms a Ubc13-MMS2 complex necessary for DNA damage repair but not for NF-κB activation, whereas Ubc13-Ube2v1 is involved in NF-κB activation, but not DNA repair (17). Ube2v1 interacts with Ubc13 through non-covalent binding to mediate formation of K63-linked polyubiquitin chains (K63Ub chains), which activate the NF-κB pathway to regulate inflammation and cancer occurrence (6,16,18). Previous research has demonstrated that Ube2v1-regulated matrix metalloproteinase-1 expression plays a key role in breast cancer cell invasion and metastasis, which requires NF-κB activation (16), and that Ube2v1 promotes CRC metastasis by mediating Sirt1 ubiquitination, which suppresses autophagy epigenetically (6).

In addition to the evidence provided by Shen *et al* (6), who demonstrated that Ube2v1 promoted CRC metastasis, and the evidence from the study by Wu *et al* (19), which demonstrated that Ube2v1 promoted breast cancer metastasis, Ren *et al* (8) found that a high expression of Ube2v1 was associated with the poor prognosis of patients with cervical cancer, and Dikshit *et al* (20) demonstrated that the silencing of Ube2v1 reduced malignant melanoma growth. All these studies indicate that there is an association between Ube2v1 expression and the prognosis of patients with various cancerous tumors. Due to disagreements between two pathologists regarding the results of the immunohistochemistry of CRC and normal tissue, the present study decided to use TIMER and found that Ube2v1 expression was elevated in colon cancer tissues when compared with normal colon tissues. The results of immunohistochemistry revealed that Ube2v1 expression was associated with lymph node metastasis, and a trend towards an association with stage/invasion (pT stage) was observed. Furthermore, due to the fact that some patients refused the follow-up for various reasons during the process, the remaining number of patients who continued to be followed-up was insufficient to ensure the accuracy of the statistical results. Therefore, the method of bioinformatics was adopted to investigate the association between the expression level of UBE2V1 and the prognosis of patients with CRC. Despite multiple studies suggesting that a high expression of Ube2v1 promotes CRC metastasis and affects patient prognosis, the bioinformatics analysis in the present study demonstrated that Ube2v1 expression was not associated with the prognosis/survival of patients with CRC. It was hypothesized that this may be due to different data processing and analysis methods, different sample selection and processing procedures, experimental biases, and errors in handling samples. A recent study (21) proposed that cancer is a complex multidimensional spatiotemporal ‘unity of ecology and evolution’ pathological ecosystem. Therefore, the aforementioned contradictions may not be due to a few specific reasons and may need to be comprehensively analyzed from multiple angles. In summary, further experiments are required to validate the association between Ube2v1 and the prognosis of patients with CRC.

In conclusion, the present study found that Ube2v1 expression was higher in CRC tissues than in normal tissues, and that Ube2v1 expression was associated with lymph node metastasis. Due to the limited sample size, further studies with larger sample sizes are warranted in order to validate and improve the accuracy of the results.

## Supplementary Material

Subsequent procedures used for immunohistochemistry.

## Figures and Tables

**Table I:** Association between Ube2v1 expression and the clinicopathological characteristics of patients with colorectal cancer.

		Ube2v1 expression			
Characteristic	No. of patients	Positive	Negative	χ^2^	P-value
Sex				0.755	0.385
Male	19	9	10		
Female	18	6	12		
Age (years)				-	0.724^a^
<60	12	4	8		
≥60	25	11	14		
Location of tumor				0.359	0.549
Colon	20	9	11		
Rectum	17	6	11		
Tumor size (cm)				0.359	0.549
≥5	17	6	11		
<5	20	9	11		
Tumor differentiation				2.006	0.157
Well or moderate	20	6	14		
Poorly	17	9	8		
pT stage				-	0.056^a^
pT1-2	9	1	8		
pT3-4	28	14	14		
Vascular invasion				-	0.670^a^
Present	6	3	3		
Absent	31	12	19		
Perineural invasion				-	>0.999^a^
Present	7	3	4		
Absent	30	12	18		
Lymph node metastasis				-	**<0.001** ^a^
Present	16	12	4		
Absent	21	3	18		

Data were analyzed using the Chi-squared test or

^a^Fisher's exact test. Values shown in bold font indicate statistically significant differences (P<0.05). Ube2v1, ubiquitin-conjugating enzyme E2 variant 1.

## Data Availability

The datasets used and/or analyzed during the current study are available from the corresponding author on reasonable request.
