# Supplementary material for: Clinicopathological significance and prognostic implications of Ube2v1 expression in colorectal cancer
Source: Med Int (Lond). 2023 Oct 31;3(6):59. doi: 10.3892/mi.2023.119 (PMC10636623; doi:10.3892/mi.2023.119)
Supplement: Subsequent procedures used for immunohistochemistry. [file Supplementary_Data.pdf]

Data S1. Subsequent procedures used for immunohistochemistry.

Required instruments and equipment. A pipette (DLAB Scientific Co., Ltd.), immunohistochemistry pen (Fuzhou Maixin Biotechnology Development Co., Ltd.), drying oven (Thermo Fisher Scientific, Inc), timer (Deli Group Co., Ltd.), incubator (Fuzhou Maixin Biotechnology Development Co., Ltd.), staining rack (Fuzhou Maixin Biotechnology Development Co., Ltd.), coverslips (Citotest Labware Manufacturing Co., Ltd.), biological microscope (Olympus BX53 light microscope, Olympus Corporation), wash bottles (Fuzhou Maixin Biotechnology Development Co., Ltd.), electromagnetic stove (Guangdong Galanz Group Co., Ltd.) and pressure cooker (Guangdong Galanz Group Co., Ltd.) were used.

Preparation of solutions. The DAB staining solution was prepared prior to use. For preparation, stable DAB buffer (1 ml, Fuzhou Maixin Biotechnology Development Co., Ltd.), stable DAB substrate (50 µl, Fuzhou Maixin Biotechnology Development Co., Ltd.) and stable DAB chromogen (50 µl, Fuzhou Maixin Biotechnology Development Co., Ltd.) were sequentially added to a small test tube to prepare the DAB staining solution.

Experimental temperature conditions and steps. The experiment was carried out at room temperature. The experimental steps were as follows: i) Deparaffinization and hydration: The paraffin-embedded sections were placed in fresh xylene, soaked for 15 min twice to remove excess liquid, and then placed in absolute ethanol, followed by soaking for 3 min twice to remove excess liquid. They were then placed in 95% ethanol, followed by soaking for 3 min. The excess liquid was then removed and the sections were placed in 85% ethanol and soaked for 3 min. They were then rinsed with tap water for 1 min. ii) Antigen retrieval: The sections were immersed in antigen retrieval solution

(EnVision FLEX Target Retrieval Solution, High pH; cat. no. K8000; Agilent Technologies, Inc.) while placing the container in a pressure cooker and heating it to boiling, covering the pressure valve and maintaining the steam for 1-4 min. iii) Blocking endogenous peroxidase: The antigen-retrieved sections were rinsed with tap water for 1 min and a pen was used to circle the area of interest on the slide, followed by rinsing with PBS solution (Agilent Technologies, Inc.) for 3 min, repeating for three times. After removing the PBS solution, 100 µl endogenous peroxidase blocking agent was added within the circled area, followed by incubation at room temperature for 10 minutes, and rinsing with PBS solution for 3 min, repeating three times. iv) Addition of antibody: Following the removal of the PBS solution, add 100 µl primary antibody (Ube2v1, cat. no. E-AB-18501, Elabscience Biotechnology Co., Ltd., diluted 1:100) was added, followed by incubation at room temperature for 60 min, and rinsing with PBS solution for 3 min, repeating three times. v) Addition of reaction enhancer: Following the removal of the PBS solution, 100 µl reaction enhancer (Fuzhou Maixin Biotechnology Development Co., Ltd.) was added followed by incubation at room temperature for 20 min, and rinsing with PBS solution for 3 min, repeating three times. vi) Addition of enzyme-labeled anti-mouse/rabbit IgG polymer: The PBS solution was removed and 100 µl enzyme-labeled anti-mouse/rabbit IgG polymer (cat. no. K8000; Agilent Technologies, Inc.) were then added, followed by incubation at room temperature for 30 min, and rinsing with PBS solution, three times, for 3 min each time. vii) Staining: Following the removal of the PBS solution, 100-200 µl freshly prepared DAB staining solution were added, followed by incubation at room temperature for 3-5 min. viii) Counterstaining: The sections were rinsed with tap water, and 100-200 µl hematoxylin staining solution (Fuzhou Maixin Biotechnology Development Co., Ltd.) were then added, followed by incubation for 10-30 sec (room temperature), or staining with self-prepared hematoxylin staining solution until the cell nuclei appear light blue. The sections were then rinsed with tap water and the PBS solution turned blue. ix) Dehydration, clearing and mounting: The sections were soaked in 85% ethanol for 3 min, soaked in 95% ethanol for 3 min, soaked in absolute ethanol for 3 min, cleared with xylene, and mounted with neutral mounting medium and coverslips. The slides

were examined under a biological microscope (Olympus Corporation). x) Interpretation of results: The staining solution will precipitate in the areas where the corresponding antigen is present, resulting in a brownish-yellow substance. The staining results need to be observed and interpreted by qualified pathologists under a biological microscope after staining the tissue sections.
